# Follow-up: Prospective compound design using the ‘SAR Matrix’ method and matrix-derived conditional probabilities of activity

**DOI:** 10.12688/f1000research.6271.2

**Published:** 2015-04-15

**Authors:** Disha Gupta-Ostermann, Yoichiro Hirose, Takenao Odagami, Hiroyuki Kouji, Jürgen Bajorath

**Affiliations:** 1 Department of Life Science Informatics, B-IT, LIMES Program Unit Chemical Biology and Medicinal Chemistry, Rheinische Friedrich-Wilhelms-Universität, Bonn, D-53113, Germany; 2 PRISM BioLab Corporation, Kanagawa, 226-8510, Japan

**Keywords:** compound design, SAR matrix, structure activity

## Abstract

In a previous Method Article, we have presented the ‘Structure-Activity Relationship (SAR) Matrix’ (SARM) approach. The SARM methodology is designed to systematically extract structurally related compound series from screening or chemical optimization data and organize these series and associated SAR information in matrices reminiscent of R-group tables. SARM calculations also yield many virtual candidate compounds that form a “chemical space envelope” around related series. To further extend the SARM approach, different methods are developed to predict the activity of virtual compounds. In this follow-up contribution, we describe an activity prediction method that derives conditional probabilities of activity from SARMs and report representative results of first prospective applications of this approach.

## Introduction

In recent years, graphical methods have substantially expanded the medicinal chemistry repertoire for analyzing Structure-Activity Relationships (SARs) ^1, 2^. The development of computational techniques to visualize SAR patterns and identify key compounds has in part been catalyzed by increasing volumes and complexity of activity data in medicinal chemistry. Going beyond a purely descriptive nature of graphical SAR exploration, as exemplified by activity landscape representations ^1^, the SAR Matrix (SARM) approach ^3^ was conceptualized to combine large-scale graphical SAR analysis and compound design. SARM calculations generate many virtual compounds (VCs) that populate chemical space around structurally related series. In order to prioritize virtual candidate compounds from SARMs in a target/assay-specific manner, activity prediction methods have been developed including local Quantitative SAR (QSAR) models utilizing compound neighborhood information in SARMs ^4^ and an approach that derives conditional probabilities of activity from SARMs ^5^.

In a previous Method Article ^6^, the SARM methodology and extensions have been described including matrix-based QSAR ^4^ and navigation of multi-target activity spaces ^7^. In this follow-up contribution, we focus on a conditional probability-based approach to activity prediction, which is distinct from QSAR analysis, and report results of first prospective applications. While we are currently unable to disclose the structures of active compounds (due to patent issues of PRISM Biolab Corporation), the prediction statistics and exemplary results we report for an actual drug discovery project should be helpful to put SARM-based predictions into perspective, beyond computational benchmarking, and might spark the interest of practitioners in this field.

## Methods

Since details of the SARM methodology and matrix-based QSAR modeling have been presented in the accompanying article ^6^, we initially provide only brief summaries of these methods, followed by a detailed description of the conditional probability approach.

### SAR matrices

**Figure 1.**
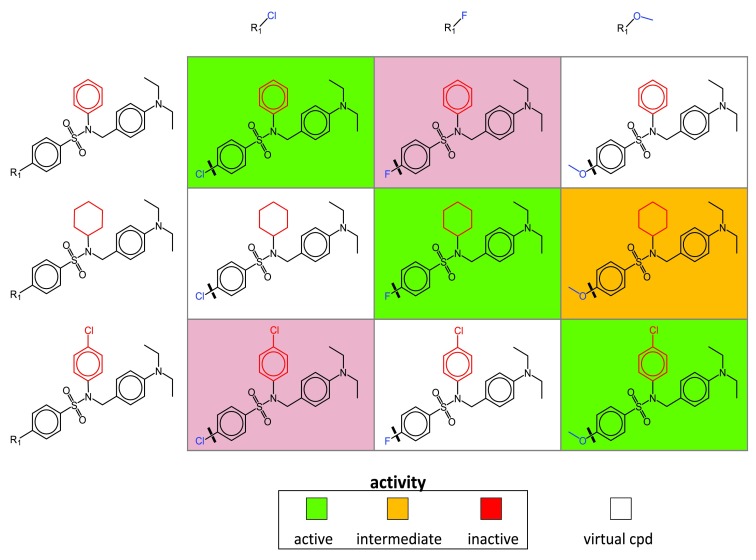
SAR matrix. A schematic representation of a SARM is shown. Compound fragmentation (indicated by thick lines in matrix cells) yields three analog series with structurally related cores (keys). Each series consists of analogs that share a core and differ by a single substituent (value, blue). Structural differences between the cores of the three series are highlighted in red. Each SARM combines all analog series with structurally related cores available in a compound set. Rows and columns represent compounds sharing the same core and substituent, respectively. In each cell, the combination of a core and a substituent defines a unique molecular structure. Compounds present in the data set are represented by filled cells that are color-coded according to activity. In addition, empty cells represent virtual compounds (i.e., previously unexplored key-value combinations resulting from MMP fragmentation).

To generate SARMs compounds are subjected to a systematic two-step fragmentation procedure yielding matched molecular pairs (MMPs) ^8^. An MMP is defined as a pair of compounds that only differ at a single site. In the first step, compounds are fragmented into core structures and substituents. In the second step, resulting core structures are subjected to fragmentation. This two-step fragmentation protocol identifies series of compounds with related core structures (forming “core MMPs”). Series of compounds with cores forming MMP relationships are organized in individual SARMs, as illustrated in Figure 1. Each matrix cell defines a unique combination of a core and substituent (reminiscent of yet distinct from R-group tables). Following MMP terminology, the core is called key fragment and the substituent value fragment ^8^. Each filled cell represents an actual compound color-coded by activity or potency and each empty cell a VC representing a previously unexplored core-substituent (key-value) combination. Accordingly, VCs are thought to generate a “chemical space envelope” around structurally related compound series. Depending on the structural relationships that are present within a given compound set, varying numbers of SARMs are obtained that systematically organize available analog series and provide many VCs for further consideration. The more similar data set compounds are to each other, the more SARMs are typically obtained.

### Matrix-based local QSAR models

**Figure 2.**
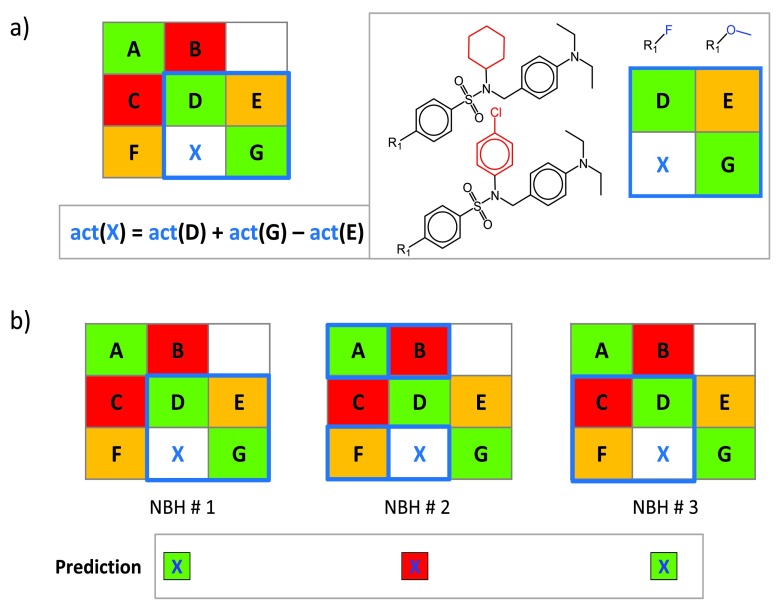
Neighborhood-based activity prediction. ( **a**) A NBH of virtual compound X is marked in blue in a model SARM and compounds forming this NBH are displayed. Compounds D and G share the same substituents and core with X, respectively, and the third neighbor E consists of the core of D and substituent of G. At the lower left, the equation to predict the potency of X from the values of D, E, and G is shown. ( **b**) The process of NBH mining is illustrated. For X, the set of all qualifying NBHs (marked in blue) in a given SARM are identified and potency values are predicted for individual NBHs (indicated by color-coded squares). “act” stands for activity (in this case, numerical potency values are used).

A compound neighborhood (NBH) approach was developed for potency prediction of VCs based on known potencies of structural analogs ^4^, as illustrated in Figure 2. A qualifying NBH consists of two known active compounds that contain the key and value fragment of a given VC, respectively (D and G in Figure 2a), and a third active compound (E) that consists of the key of D and value of G. The potency of a VC can then be predicted from its neighbors by applying the additivity assumption underlying Free-Wilson analysis ^9^ using the equation shown in Figure 2a. For a given VC, all qualifying NBHs are identified across all SARMs, as illustrated in Figure 2b, and for each NBH, an individual potency prediction is carried out using a local “mini-QSAR” model. The average potency over all NBHs is then calculated to yield the final prediction.

The NBH approach is based upon numerical values and thus well suited for potency prediction during compound optimization considering multiple analog series. Principal limitations of QSAR modeling also apply to the NBH methodology, given its Free-Wilson foundation. Hence, meaningful potency predictions can only be expected in the presence of SAR continuity (when small structural changes are accompanied by gradual changes in potency). By contrast, SARMs capturing discontinuous SARs or activity cliffs ^10^ fall outside the QSAR applicability domain. Because potency predictions are carried out over multiple NBHs in different SARMs, standard deviations of predictions provide a simple yet effective indicator of prediction reliability. High and low standard deviations indicate the presence of SAR discontinuity and continuity, respectively, for compound subsets involved in the predictions. When standard deviations are low, accurate SARM-based potency predictions can be expected ^4^.

### Predictions based on conditional probabilities of activity

**Figure 3.**
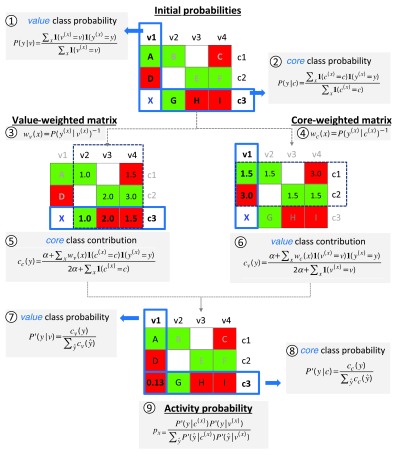
Predictions based on conditional probabilities of activity. Steps and equations required to derive probabilities of activity from SARMs for prediction of virtual compound X are shown using a model SARM with nine compounds (A–I) that contain three cores ( *c1*– *c3*) and four values ( *v1*– *v4*). Matrix cells are color-coded according to compound activity (red, inactive; green, active). In the first step, initial class probabilities are calculated for all cores and values using equation 2 and 1, respectively. Value- and core-weighted matrices are then derived via equations 4 and 3. The class contribution of core *c3* is obtained from the value-weighted matrix using equation 5. Analogously, the class contribution of value *v1* is obtained from the core-weighted matrix using equation 6. The value and core class contributions are then normalized using equations 7 and 8. Finally, the activity probability *p _x_* of 0.13 is obtained for X by combining the normalized core *c3* and value *v1* class probabilities using equation 9.

A conceptually different approach was developed for hit expansion from screening data based upon conditional probabilities of activity derived from SARMs, as outlined in Figure 3. In contrast to NBH-based prediction of numerical potency values, the conditional probability method can utilize approximate potency measurements (e.g., primary screening data) leading to a binary classification of inactive vs. active data set compounds and ensuing prediction of a probability of activity for VCs.

The conceptual basis of the approach is provided by the following ideas: based on the observed frequency of occurrence of given core and value fragments in active versus inactive compounds (in the following referred to as the active versus inactive class), probabilities of activity and inactivity can be derived for cores and values. Importantly, the contributions of cores and values are thought to be influenced by each other because compounds are represented in SARMs as combinations of individual core and value fragments. Considering the conditional nature of core and value contributions to activity, initial probabilities are weighted to derive class probabilities for any core and value. For a given VC, probabilities of its core and value are then combined to yield a final probability of activity.

Key steps of the methodology are summarized in Figure 3 (and for each step, the respective equation is provided). To illustrate the approach in an intuitive manner, we will go through an exemplary probability calculation for a given VC, guided by Figure 3.

### Core and value class probabilities

The SARM in Figure 3 contains nine compounds (A–I) that comprise three cores (c1–c3) and four values (v1–v4). The probability of activity will be predicted for virtual compound X that shares core *c3* with compounds G, H, and I and value *v1* with compounds A and D.

Given the distribution of individual values *v* and cores *c* in active and inactive compounds, probabilities of activity and inactivity are calculated using equation 1 and equation 2. Here, *P*( *y*| *v*) and *P*( *y*| *c*) are the conditional probabilities that describe how likely it is to observe a given specific class *y* ∈{active, inactive} for a value *v* and a core *c*, respectively. If *c* ^*(x)*^, *v* ^*(x)*^, *y* ^*(x)*^ is the core, value, and class of a given compound *x*, we can express the conditional probabilities as the fraction of compounds with a core *c* or value *v* and class *y* relative to all compounds containing this core or value. In case of value *v1*, both class probabilities are equal (i.e., 1/2) because *v1* is contained in one active and one inactive compound. By contrast, the probability of inactivity is two times higher for core *c3* than its probability of activity (2/3 vs. 1/3).

### Core- and value-weighted matrices

These initial estimates are further refined by taking information from all SARM compounds into account. For this, the inverse of value and core class probabilities is used to derive the *value-weighted matrix* and *core-weighted matrix*, respectively. In case of the value-weighted matrix, the inverse class probabilities of the values are mapped to the compounds that represent the corresponding value and class. Analogously, the core-weighted matrix is derived by mapping the inverse class probabilities of the cores to the compounds that represent the corresponding core and class. The value-weighted matrix results from the assignment of a weight to each compound using equation 3 and the core-weighted matrix is obtained using equation 4.

### Refinement of core and value class contributions

In this step, core probabilities using value-weighted matrices and value probabilities using core-weighted matrices are derived. The underlying idea is to statistically assess if a core or value contributes more to activity or inactivity. This rationalizes the calculation of weights from the previous step: the less frequently observed class for a core or value is assigned a higher weight, which leads to a larger class contribution of the corresponding value or core of a compound, respectively. For example, the class probability of core *c3* is updated by considering information from values *v2*, *v3*, and *v4* in compound G, H, and I, respectively. All compounds containing value *v2* are active (2/2); hence, the core class probabilities of compounds B and G are assigned a weight of 1.0 (through value-weighting). For value *v3*, the compounds show equal class frequency of (in)activity (1/2); thus, both active and inactive compounds are assigned the same weight of 2.0. Finally, two of three compounds containing value *v4* are inactive. Accordingly, inactive compound I receives a lower weight of 1.5 indicating that its inactivity is more likely due to *v4*. It follows that with increasing frequency of inactivity for a given value, core weights of inactive compounds decrease (and *vice versa*), indicating that the value is likely to be responsible for inactivity. Analogous considerations apply to assess probabilities of activity.

From the value-weighted matrix, core class contributions are calculated with equation 5. For core *c3*, contributions of 0.34 and 1.12 to activity and inactivity are obtained, respectively, using a smoothing factor of α=0.1 (this factor is applied to prevent zero probabilities when no compound is available to represent a possible core or value class):

$$C_{c3}(act)=\frac{0.1+1.0}{0.2+3}=0.34$$

$$\;\;C_{c3}(inact)=\frac{0.1+2.0+1.5}{0.2+3}=1.12$$

Through normalization using equation 7 core class probabilities between 0 and 1 are obtained; for *c3* values of 0.23 (activity) and 0.76 (inactivity).

Analogously, value class probabilities are refined using the core-weighted matrix (generated using equation 4). For example, class probabilities of value *v1* are adjusted by considering information from cores *c1* and *c2* in compounds A and D that contain *v1*. Compound A is active and belongs to the majority class of *c1* and is thus assigned a lower weight than D, which is inactive and belongs to the minority class of *c2*. The higher weight assigned to compound D means that its inactivity is statistically more likely to result from value *v1* than core *c2*. Weighted value class contributions calculated using equation 6 give activity and inactivity contributions of 0.72 and 1.40, respectively, for value *v1* (applying a smoothing factor of α=0.1):

$$\;C_{v1}(act)=\frac{0.1+1.5}{0.2+2}=0.72$$

$$C_{v1}(inact)=\frac{0.1+3.0}{0.2+2}=1.40$$

Normalization using equation 8 then yields updated *v1* class probabilities of 0.34 (activity) and 0.66 (inactivity).

### Combined activity probability

Finally, the normalized core and value probabilities are combined via equation 9 yielding an activity probability *p _x_* (ranging from 0 to 1) for any core-value combination representing a VC. Increasing *p _x_* values indicate an increasing probability of activity. For classification, a threshold value of activity must be set (e.g., 0.5). In our example, the normalized core and value class probabilities for *c3* and *v1* result in an activity probability *p _x_* of 0.13 for virtual compound X representing this core-value combination. Thus, given the low probability of activity, this VC is predicted to be inactive. In benchmark calculations on sets of known active and inactive compounds, conditional probability calculations yielded reasonably accurate predictions of activity, at least comparable to current state-of the-art machine learning approaches ^5^.

Because the conditional probability approach is statistically grounded, prediction accuracy is expected to increase with sample sizes and matrix density ^6^. Therefore, it makes sense to exclude SARMs from the calculations that contain only a small number of data set compounds or have limited row overlap (accounting for shared substitution patterns among structurally related series) ^6^. Accordingly, SARMs with more than 50% row overlap are typically considered informative and prioritized for probability calculations.

Different from the NBH approach, the conditional probability method is generally applicable and not confined to compound subsets representing continuous SARs. Thus, QSAR applicability domain restrictions do not apply in this case.

## Application

**Figure 4.**
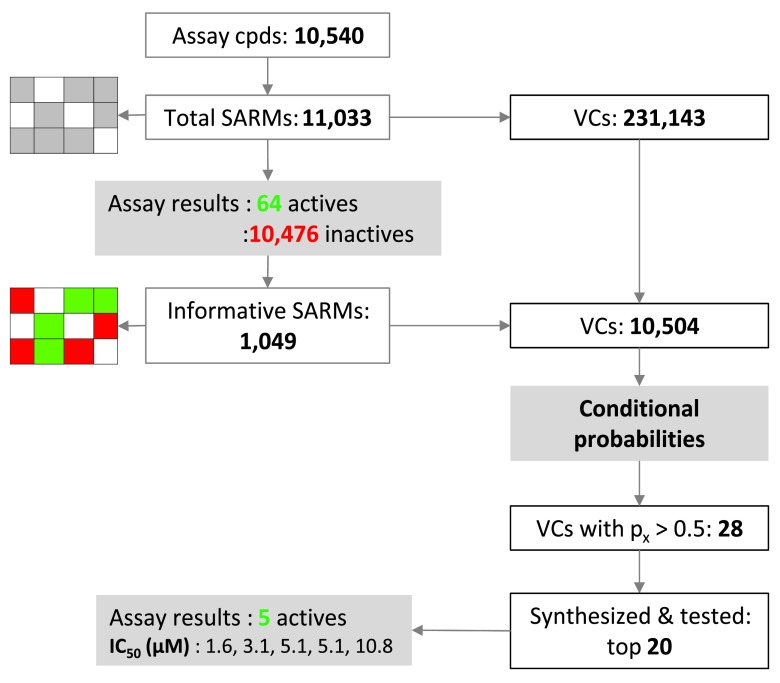
SAR matrix and prediction statistics. SAR matrix statistics for a library of alpha helical turn mimetics are provided and activity predictions for virtual compounds are summarized. For these predictions, conditional probabilities of activity were derived from library screening data.

The conditional probability method has been used for activity predictions (hit expansion) starting from the results of a screen of the PRISM library of alpha helical turn mimetics ^11, 12^ carried out in search of new inhibitors of the Wnt/β-catenin protein-protein interaction and pathway ^13, 14^. The Wnt pathway is implicated in a variety of disease states including several forms of cancer. Consequently, inhibitors of the Wnt/β-catenin interaction are thought to have high therapeutic potential ^13, 14^. PRISM’s current helix mimetics library contains more than 10,000 small molecules with closely related scaffolds ^11, 12^ suitable for SARM analysis. These compounds are analogs containing closely related scaffolds with three substitution sites each. The library screen was carried out using a luciferase reporter gene assay of the Wnt pathway ^15, 16^ and the stably transfected cell line Hek-293, STF1.1 ^11^. Figure 4 summarizes SARM analysis of the library and activity predictions. The library contained a total of 10,540 compounds that yielded 11,033 stereochemistry-sensitive SARMs (i.e., matrices explicitly accounting for all stereoisomers) with a total of 231,143 VCs. This matrix distribution was solely determined by structural relationships between library compounds.

Screening of the library in the reporter gene assay yielded 64 active and 10,476 inactive compounds (applying a threshold of less than 50% residual luciferase activity). Hence, only a limited number of compounds were classified as active applying this threshold. Active and inactive compounds were then mapped to SARMs and a subset of 1,049 informative matrices (with at least 50% row overlap) was selected that contained 10,504 VCs. Probability calculations predicted 28 VCs to be active. Twenty candidates were synthesized, re-screened, and tested in confirmatory assays, leading to the identification of five novel hits with activities in the low-micromolar range. These five novel actives were, by design, analogs of library compounds having previously unconsidered substitution patterns involving two different sites.

## Data availability

In a deposition on the open access ZENODO platform ^17^, the following data have been made available. Detailed probability calculations for the matrix in Figure 3 are provided in an excel sheet. Furthermore, SARMs generated from the PRISM library on which the calculations were based are made available without compound structures (compounds are represented by unique identification). On the basis of these SARMs, the predictions can be fully reproduced.

## Concluding remarks

In this contribution, we have discussed methodological advances for activity prediction on the basis of SARMs, which systematically account for structural/analog relationships in compound sets of any source, organize structurally related compound series, and yield virtual candidate compounds. In combination with the SAR matrix method, compound neighborhood analysis based upon Free-Wilson principles and derivation of conditional probabilities of activity are applicable to predict novel active compounds at different stages of chemical optimization efforts. The conditional probability approach detailed herein is particularly suitable for hit expansion and can be applied to raw screening data. Going beyond benchmark calculations, first prospective applications have yielded promising results. For example, screening data of the PRISM library of helix mimetics made it possible to prioritize a small number of candidate compounds for synthesis from a pool of ~10,000 pre-selected VCs on the basis of only 64 preliminary screening hits. These predictions ultimately resulted in the identification of five new active compounds by considering only 20 candidates. These compounds provide new starting points for chemical optimization efforts. Of course, further prospective validation studies will need to be performed to better understand the performance of SARM-based activity predictions for different compound classes, targets, and screening assays. However, considering the well-defined scaffold-substituent patterns of compounds representing alpha helical turn mimetics and the systematic design of the library, which plays into the strength of the SARM approach, successful activity predictions are also anticipated for library screens using assay systems and targets engaged in other therapeutically relevant protein-protein interactions.

## References

[1] Wassermann AM Wawer M Bajorath J: Activity landscape representations for structure-activity relationship analysis. *J Med Chem.* 2010;53(23):8209–8223. 10.1021/jm100933w 20845971

[2] Stumpfe D Bajorath J: Methods for SAR visualization. *RSC Adv.* 2012;2(2):369–378. 10.1039/C1RA00924A

[3] Wassermann AM Haebel P Weskamp N: SAR matrices: automated extraction of information-rich SAR tables from large compound data sets. *J Chem Inf Model.* 2012;52(7):1769–1776. 10.1021/ci300206e 22657271

[4] Gupta-Ostermann D Shanmugasundaram V Bajorath J: Neighborhood-based prediction of novel active compounds from SAR matrices. *J Chem Inf Model.* 2014;54(3):801–809. 10.1021/ci5000483 24593807

[5] Gupta-Ostermann D Balfer J Bajorath J: Hit expansion from screening data based upon conditional probabilities of activity derived from SAR matrices. *Mol Inf.* 2015;34(2–3):134–146. 10.1002/minf.201400164 27490036

[6] Gupta-Ostermann D Bajorath J: The ‘SAR Matrix’ method and its extensions for applications in medicinal chemistry and chemogenomics [v2; ref status: indexed, http://f1000r.es/3rg]. *F1000Res.* 2014;3:113. 10.12688/f1000research.4185.2 25383183 PMC4215758

[7] Gupta-Ostermann D Hu Y Bajorath J: Systematic mining of analog series with related core structures in multi-target activity space. *J Comput Aided Mol Des.* 2013;27(8):665–674. 10.1007/s10822-013-9671-5 23975272

[8] Hussain J Rea C: Computationally efficient algorithm to identify matched molecular pairs (MMPs) in large data sets. *J Chem Inf Model.* 2010;50(3):339–348. 10.1021/ci900450m 20121045

[9] Kubinyi H: Free Wilson analysis. Theory, applications and its relationships to Hansch analysis. *Quant Struct-Act Relat.* 1988;7(3):121–133. 10.1002/qsar.19880070303

[10] Stumpfe D Bajorath J: Exploring activity cliffs in medicinal chemistry. *J Med Chem.* 2012;55(7):2932–2942. 10.1021/jm201706b 22236250

[11] Kouji H Kogami Y Odagami T: Alpha Helix mimetic compositions for treating cancer and other CBP/catenin-mediated diseases and conditions. US 8691819 B2,2014 Reference Source

[12] Odagami T Kogami Y Kouji H: Alpha helix mimetics and methods thereto. WO 2010128685 A1, 2010; US 20120088770 A1,2012 Reference Source

[13] Moon RT Kohn AD De Ferrari GV: WNT and beta-catenin signalling: diseases and therapies. *Nat Rev Genet.* 2004;5(9):691–701. 10.1038/nrg1427 15372092

[14] Klaus A Birchmeier W: Wnt signalling and its impact on development and cancer. *Nat Rev Cancer.* 2008;8(5):387–398. 10.1038/nrc2389 18432252

[15] Molenaar M van de Wetering M Oosterwegel M: XTcf-3 transcription factor mediates beta-catenin-induced axis formation in Xenopus embryos. *Cell.* 1996;86(3):391–399. 10.1016/S0092-8674(00)80112-9 8756721

[16] Veeman MT Slusarski DC Kaykas A: Zebrafish prickle, a modulator of noncanonical Wnt/Fz signaling, regulates gastrulation movements. *Curr Biol.* 2003;13(8):680–685. 10.1016/S0960-9822(03)00240-9 12699626

[17] Gupta-Ostermann D Hirose Y Odagami T: Follow-up: Prospective compound design using the ‘SAR Matrix’ method and matrix-derived conditional probabilities of activity. *Zenodo.* 2015 Data Source 10.12688/f1000research.6271.2 PMC4406192 25949808

