# Supplementary material for: Follow-up: Prospective compound design using the ‘SAR Matrix’ method and matrix-derived conditional probabilities of activity
Source: F1000Res. 2015 Apr 15;4:75. Originally published 2015 Mar 23. [Version 2] doi: 10.12688/f1000research.6271.2 (PMC4406192; doi:10.12688/f1000research.6271.2)
Supplement: Peer review history

# Peer review: Referee response for version 2

**DOI:** 10.5256/f1000research.6835.r8335

**Published:** 2015-05-14

**Authors:** Dragos Horvath

**Affiliations:** 1 Laboratoire de Chémoinformatique and Laboratoire d'Infochimie, UMR 7140 CNRS (LCS), CNRS-Université de Strasbourg, Strasbourg, France

My thanks to the authors for having followed up my previous suggestions - I am satisfied now.

I have read this submission. I believe that I have an appropriate level of expertise to confirm that it is of an acceptable scientific standard.

# Peer review: Referee response for version 1

**DOI:** 10.5256/f1000research.6727.r8067

**Published:** 2015-04-08

**Authors:** Dragos Horvath

**Affiliations:** 1 Laboratoire de Chémoinformatique and Laboratoire d'Infochimie, UMR 7140 CNRS (LCS), CNRS-Université de Strasbourg, Strasbourg, France

This is an interesting upgrade of the SARM methodology, now endeavored with a probability-driven activity prediction tool described in this paper. Unfortunately, I cannot recommend indexation as is, because the methodology is not comprehensively described: some formulae embedded in a Figure are never rigorously explained, except by means of some hand-waiving example. Therefore, I (hope I) got the principle of the method - looks very much like naive Bayes to me. If so - does it do better than standard naive Bayes, with some fragment count descriptors? Honestly, I could not write a piece of code implementing it on the basis of what is said in the paper. Don't understand cryptic annotations like 1(v(x)=v) - suppose it's some Kronecker delta symbol 'sum only over lines with v(x)=v'... but, by the way, what is 'x'? Molecules? Matrix columns? Rows? This is the best-kept secret of the publication...

I have read this submission. I believe that I have an appropriate level of expertise to confirm that it is of an acceptable scientific standard, however I have significant reservations, as outlined above.

# Response

**Published:** 2015-04-09

**Authors:** Jürgen Bajorath

**Affiliations:** Department of Life Science Informatics, B-IT and LIMES Institutes, Rheinische Friedrich-Wilhelms-Universität Bonn, Germany

The SARM-based probability approach and Naïve Bayesian (NB) classification are both probabilistic in nature and attempt to estimate the posterior probability of a given compound *x* to belong to a class *y, *i.e. *P*( *y *| *x*). However, following Bayes’ theorem, NB modeling derives the posterior from the prior and class likelihood. The class likelihood, *P*( *x *| *y*), is estimated from the data. By contrast, the SARM-based approach assigns weights to the cores (keys) based on substituents (values) and vice versa. This is done under the assumption that either cores, values, or their combination might be responsible for the activity. Thus, the approach estimates the posterior from the data and then applies a re-weighting (refinement) scheme by calculating core and value class contributions.

All methodological details of the SARM-based probability of activity approach are provided in reference 5 of the paper.

# Peer review: Referee response for version 1

**DOI:** 10.5256/f1000research.6727.r8160

**Published:** 2015-03-31

**Authors:** Stefan Laufer

**Affiliations:** 1 University of Tübingen, Tübingen, Germany

This Method article mostly describes an extension of the SAR Matrix approach to predict active compounds from many virtual candidates that are contained in matrices derived from compound libraries.

The new activity prediction method is generally applicable to screening data to facilitate hit expand and can make use of approximate activity measurements such as % inhibition. This would be attractive in practice.

The conditional probability method, which was first published in an informatics journal, is not trivial and probably not easy to understand for many medicinal chemists.

Therefore, the authors were obviously motivated to make this prediction methodology accessible to wider audience in screening and medicinal chemistry. They have done so by going step by step through exemplary calculations that illustrate ideas behind this approach and show how active compounds are predicted.

In addition, they report first practical applications that should make this method in combination with the SAR matrix structure attractive to many.

Although the application on a library of helix mimetics is essentially proprietary (structures of active compounds cannot be shown), the statistics of the predictions are interesting. Of thousands of virtual compounds the SAR matrix approach generates for this library, only 28 were predicted to be active using the new method when processing reporter gene assay data probing the Wnt pathway. Twenty of these compounds were synthesized and tested and 5 new hits were identified with low-micromolar potency. Clearly, if the combined SAR matrix / activity prediction approach produces similar results in additional applications of this library or other screening libraries, it will be

rather useful for hit expansion.

Taken together, the authors have attempted to make a relatively complex computational approach easier to appreciate by a screening or chemistry audience by providing easy to follow examples and practical applications. This could hardly be accomplished in a specialized computational journal.

I have read this submission. I believe that I have an appropriate level of expertise to confirm that it is of an acceptable scientific standard.

# Response

**Published:** 2015-04-09

**Authors:** Jürgen Bajorath

**Affiliations:** Department of Life Science Informatics, B-IT and LIMES Institutes, Rheinische Friedrich-Wilhelms-Universität Bonn, Germany

We thank the reviewer for pointing at a primary motivation for this publication and for emphasizing accessibility to a wider than an expert computational audience.

# Peer review: Referee response for version 1

**DOI:** 10.5256/f1000research.6727.r8069

**Published:** 2015-03-31

**Authors:** Georgia B. McGaughey

**Affiliations:** 1 Vertex Pharmaceuticals Inc., Cambridge, MA, USA

Gupta-Ostermann's "follow-up" manuscript is well written and clearly laid out. I only have a few (minor) recommendations, which I believe would help readers more easily replicate their work.

The added value of this manuscript lies in figure 3 where "conditional probabilities of activity" are explained. The authors have explained conditional probabilities with figures, text and associated mathematical equations and have even gone so far as to carry out the math for the weighted core class contributions. For interested readers who want to implement the conditional probabilities concept in their own research, I highly suggest that real (or toy) data be included, in the very least, as supplemental material with all the data completely worked out, not just the weighted core class contributions. This would allow one to implement the concept, carry out the math and compare the results to the published results more easily. Additionally, although text is included to explain conditional probabilities, I found myself having to read this section a few times to fully understand the clear impact this method could have. I think this section needs to be expanded with more text.

Finally, although it is understandable that the work carried out herein with PRISM BioLab Corporation, is proprietary, it is unfortunate that more information regarding the "twenty synthesized candidates" can not be elaborated upon.  Any information regarding the similarity of these compounds to the actives (or even the similarity range of the actives themselves) would be informative.

I have read this submission. I believe that I have an appropriate level of expertise to confirm that it is of an acceptable scientific standard.

# Peer review: Referee response for version 1

**DOI:** 10.5256/f1000research.6727.r8159

**Published:** 2015-03-31

**Authors:** Hans Matter

**Affiliations:** 1 Design and Informatics, Sanofi-Aventis Deutschland GmbH, Frankfurt am Main, Germany

This interesting contribution by Bajorath *et al. *nicely extends the idea of graphical methods for SAR analysis in computational medicinal chemistry. The SARM method was shown earlier to capture SAR information from larger collections by matched molecular pairs (MMPs) and to present it in an intuitive way. Furthermore the combination of large-scale SAR analysis with virtual compounds allows guiding synthesis to explore straightforward ideas as direct outcome of SAR interpretation. Therefore this approach is attractive to rapidly identify activity trends and cliffs.

The paper reports a conditional probability-based approach to activity prediction from SAR knowledge. Such a conditional probability measures the probability of activity for one compound given that a structurally related compound was active. Individual probabilities are extracted from rows and columns in the underlying SARMs. While such a probabilistic approach only works for SARMs, which are sufficiently populated and have shared substitution pattern, the approach is not restricted to compound subsets representing continuous SAR only.

The prospective application of this interesting concept suffers from the lack of chemical structures, so that the degree of similarity between actives and follow-up design cannot be assessed. Furthermore the description of the HTS assay, substructure alerts, additional filtering, assay validation and retesting rates, compound QCs for actives is missing. This makes it difficult to evaluate the true HTS outcome using potentially noisy data for such a challenging PPI target.

To illustrate the value of the novel activity estimation approach from matrices, it might be useful constructing a standard 2D-QSAR model and check is for predictivity of the synthesized top-20 design proposals in comparison to the matrix-derived conditional probability. It might be of interest to see, how robust both approaches work with noisy primary screening data.

The manuscript title and abstract cover the content well. The chemoinformatics approach is clearly described and can most likely be reproduced. As this is not the case for the HTS actives and the assays for this study, the results will be difficult to reproduce. The authors might also want to mention, whether software tools from their study are available to the public.

I have read this submission. I believe that I have an appropriate level of expertise to confirm that it is of an acceptable scientific standard.

# Response

**Published:** 2015-04-09

**Authors:** Jürgen Bajorath

**Affiliations:** Department of Life Science Informatics, B-IT and LIMES Institutes, Rheinische Friedrich-Wilhelms-Universität Bonn, Germany

A conventional QSAR model has been difficult to derive in this case because of the rather approximate nature of activity annotations obtained from raw screeing data. Instead, a cross-validated binary QSAR model has been generated from the screening data using the Molecular Operating Environment (version 2013.08; Chemical Computing Group Inc., Montreal, Canada) and applied to predict the activity state of the 20 test compounds, producing an accuracy of 0.65 for active and inactive compounds.
